# The professional culture among physicians in Sweden: potential implications for patient safety

**DOI:** 10.1186/s12913-018-3328-y

**Published:** 2018-07-11

**Authors:** Marita Danielsson, Per Nilsen, Hans Rutberg, Siw Carlfjord

**Affiliations:** 1 Department of Medical and Health Sciences, Division of Health Care Analysis, Linköping University, SE-581 83 Linköping, Sweden; 2 Head Office, County Council of Östergötland, SE-581 91 Linköping, Sweden; 3 Department of Medical and Health Sciences, Division of Community Medicine, Linköping University, SE-581 83 Linköping, Sweden

**Keywords:** Physicians, Patient safety, Safety culture, Qualitative research

## Abstract

**Background:**

Patient safety culture, i.e. a subset of an organization’s culture, has become an important focus of patient safety research. An organization’s culture consists of many cultures, underscoring the importance of studying subcultures. Professional subcultures in health care are potentially important from a patient safety point of view. Physicians have an important role to play in the effort to improve patient safety. The aim was to explore physicians’ shared values and norms of potential relevance for patient safety in Swedish health care.

**Methods:**

Data were collected through group and individual interviews with 28 physicians in 16 semi-structured interviews, which were recorded and transcribed verbatim before being analysed with an inductive approach.

**Results:**

Two overarching themes, “the competent physician” and “the integrated yet independent physician”, emerged from the interview data. The former theme consists of the categories Infallible and Responsible, while the latter theme consists of the categories Autonomous and Team player. The two themes and four categories express physicians’ values and norms that create expectations for the physicians’ behaviours that might have relevance for patient safety.

**Conclusions:**

Physicians represent a distinct professional subculture in Swedish health care. Several aspects of physicians’ professional culture may have relevance for patient safety. Expectations of being infallible reduce their willingness to talk about errors they make, thus limiting opportunities for learning from errors. The autonomy of physicians is associated with expectations to act independently, and they use their decisional latitude to determine the extent to which they engage in patient safety. The physicians perceived that organizational barriers make it difficult to live up to expectations to assume responsibility for patient safety. Similarly, expectations to be part of multi-professional teams were deemed difficult to fulfil. It is important to recognize the implications of a multi-faceted perspective on the culture of health care organizations, including physicians’ professional culture, in efforts to improve patient safety.

## Background

Patient safety culture has become an important focus of patient safety research. There are emerging empirical research findings to support the effectiveness of interventions targeting patient safety culture for improving patient safety [1]. Patient safety culture is typically viewed as a subset of organizational culture, i.e. those aspects of the organizational culture that influence patient safety [2, 3]. There are many definitions of culture [2]; a commonly cited example is that culture is the way we do things around here. Most definitions of culture highlight the importance of the values and norms that are shared among members of a social group, e.g. a team, profession or organization, and which create expectations for certain behaviours. Values are principles that guide the behaviours of the members of a social group, serving as a glue to hold them together [4]. The values shape the norms, which are unwritten rules for behaviours that are expected by the members of the social group [5].

Organizations are usually considered to be multi-cultural given the wide variety of professional groups, departments, divisions and teams operating within them, underscoring the importance of studying subcultures to develop a deeper understanding of the prevailing culture in an organization. Studies suggest that patient safety cultures can differ between departments, specialties and professional groups [1, 6, 7]. Subcultures emerge in groups among colleagues with similar educational backgrounds who are in frequent interaction, perceive themselves to be distinct from other groups in the organization and share similar problems and in-group understandings of ways of solving such problems [8].

Differences between the professional cultures of physicians and nurses are often described in terms of physicians being trained to take charge and assume a role of leadership and responsibility for decisions, whereas nurses are more trained to work in teams and collectively work out problems [9]. In the area of patient safety, research has shown that physicians and registered nurses differ with regard to their values and norms concerning adverse events reporting [10, 11].

Professional subcultures in health care are potentially important from a patient safety point of view. Patient safety may be compromised if the subcultures are not aligned with the organization-wide patient safety goals or if different values and norms hinder effective communication, learning or teamwork across professions. In a previous study of registered nurses and nurse assistants, we found notable differences concerning patient safety-related values and norms among the two professional groups [12]. A better understanding of the subcultures may facilitate the use of more appropriate or tailored efforts to influence the patient safety culture and improve patient safety. More qualitative research has been called for to gain improved understanding of patient safety culture and professional culture [13, 14].

Although physicians have an important role to play in the effort to improve patient safety, some studies have shown that they tend to be less motivated to participate in quality improvement efforts than other professionals [15], are reluctant to engage fully in inter-professional teamwork [16] and are sceptical of the value of adverse event reporting [17] and various forms of standardization, e.g. protocols and reminders [16]. The extent to which such barriers to safer care can be explained with reference to behaviours that are associated with physicians’ professional culture has not been explored. We have not been able to find any previous studies that have investigated whether physicians hold values and norms that influence their behaviours in such a way that patient safety may be compromised. Therefore, the aim of this study was to explore physicians’ shared values and norms of potential relevance for patient safety in Swedish health care.

## Methods

### Study setting

The study was carried out in Sweden. Health care in Sweden is mainly publicly funded although private health care also exists. All citizens are insured by the state, with equal access for the entire population. Out-of-pocket fees are low and regulated by law. The provision of health care services in Sweden is primarily the responsibility of the 21 county councils throughout Sweden. The health care system is financed mainly through taxes levied by county councils and municipalities.

### Participants

The study population consisted of three categories of physicians: interns, residents and consultants. The physicians were employed in medical and surgical wards at two hospitals in southeast Sweden. In total, 28 physicians participated in 16 interviews of which 6 were group interviews and 10 were individual interviews (Table 1).

**Table 1:** Characteristics of the informants

Characteristics	Interns (*n* = 11)	Residents (*n* = 11)	Consultants (*n* = 6)
Sex, *n*			
Male	6	6	3
Female	5	5	3
Experience in profession, months	4–17	6–58	6–108
Group/individual interviews, *n*	4/0	2/4	0/6
Medicine/surgery, *n*	Not specified	8/3	2/4

Physicians’ education in Sweden encompasses 5.5 years of theoretical studies followed by 2 years of internship and approximately 5 years of residency to become a resident. After a further period of clinical experience, the physician can apply for a position as a consultant.

The interns were recruited by means of information sent to their manager (all interns belong to one unit in the organization during their internship) with a request to invite four to six interns for an interview on patient safety. The residents and consultants were invited by the head of department who was informed by the author. The participants who volunteered to participate were then individually informed about the study purpose.

### Data collection

This qualitative study used an inductive approach, using topics rather than questions, to allow the participants to talk and reflect about patient safety to gain insights into their values and norms of relevance for this area. This allowed detailed data to be gathered with flexibility and follow-up of interesting lines of inquiry. The topics consisted of themes related to patient safety.

Two forms of interviews were conducted. Group interviews [18] were conducted with all interns. Two group interviews and four individual interviews were conducted with the residents. All interviews with the consultants were individual. Individual interviews were conducted when the work situation did not permit group interviews. In total, 6 group interviews and 10 individual interviews were conducted.

Each interview began with an open, introductory question, “What is patient safety and what does it mean to you?” The interview focused on (a) perceptions of responsibility from a patient safety perspective, “What are your thoughts about your profession’s responsibility for patient safety at the unit?” (b) situations where mistakes are made, “What happens when a mistake is made?” and (c) concerns about patient care “Tell me about your concerns regarding patient safety”. To gain deeper insights, questions were complemented with probes.

All interviews were conducted between May 2013 and November 2015. The interviews were held in a room near the ward where the study participants worked and lasted between 20 and 51 min. The first author (MD) conducted all interviews.

### Data analysis

All interviews were recorded and transcribed verbatim. Qualitative content analysis as described by Graneheim and Lundman [19] was applied for the analysis of the data. The analysis was conducted in several steps with the aim of identifying physicians’ shared values, norms and assumptions of potential relevance for patient safety. The first author (MD) listened to the recordings to ensure that the transcripts were accurate and identified meaning units. Then two authors (SC and PN) read the meaning units to obtain an understanding of the content. Several sessions were held to compare and discuss the content and label the meaning units with codes. Codes were used to sort the meaning units into subcategories based on similarity of content. The subcategories were grouped into categories, and finally themes covering the underlying content were identified. Discussions among the authors were held until consensus was reached [19] to prevent researcher bias and strengthen the internal validity*.*

Representative quotes were selected and translated from Swedish to English. In the Results, […] indicates that words are omitted from the quotation, author comments to clarify quotations appear in brackets [], and … indicates hesitation. The letter and number attached to the quotes represent intern (I), resident (R) or consultant (C) and interview number.

## Results

Two overarching themes, “the competent physician” and “the integrated yet independent physician”, emerged from the interpretation of the interview data. The former theme consists of the categories Infallible and Responsible, while the latter theme consists of the two categories Autonomous and Team player. The two themes and four categories express physicians’ values and norms that create expectations for the physicians’ behaviours that might have relevance for patient safety (Table 2).

**Table 2:** Themes and categories presented in the results section

Theme	Category
The competent physician	Infallible
	Responsible
The integrated, yet independent physician	Autonomous
	Team player

### Theme: The competent physician

The competent physician theme concerns physicians’ shared values and norms that are associated with expectations of being a highly competent professional who is infallible and is responsible for patient safety.

#### Infallible

The physicians described values and norms associated with expectations of being flawless and never committing any errors, which the physicians experienced as something of a burden. They were aware that such expectations could negatively affect physicians’ willingness to have open dialogue about mistakes, slips and lapses they make, thus restricting potential learning from errors.

The values and norms related to the highly competent and almost omnipotent physician appear to be present from the outset of medical training. During training, physicians are expected to push their own boundaries in order to develop and hone their skills. These expectations were predominantly expressed by the interns and residents.

I don’t think it’d be a problem if you ask for more help. But we’re brought up with the idea that in order to progress, you need to test your limits, from day one of training. The problem is that limits are different for different people. (R, 10).

… being a physician you should somehow be able to do most things and not make any mistakes. I think you can call that some kind of general perception. And when there’s an idea that you shouldn’t make mistakes, you probably wouldn’t tell when you have done so either, I think. (I, 1).

The physicians expressed that increased confidence over time can influence willingness to admit and talk about errors they had committed. They noted that physicians with less experience tended to be less open about things that have gone wrong, as they may feel ashamed that they do not live up to the values and norms of the profession. The physicians also mentioned positive experiences from situations when more experienced colleagues had talked about errors they had been involved with.

If you’re newly graduated, you’re a little unsure of your role. But I think that as a consultant, maybe you’re more confident, you know that you can handle some issues, and then you can talk about the things that you did wrong. Perhaps you’re more comfortable in your role as a consultant. .... It often feels good, I think, to hear other physicians talk about their mistakes. (I, 1).

First, I’m ashamed [when a mistake is done] and then I try to ignore it and I don’t want to tell anyone. Then I pull myself together and make an incident report and talk to the patient. (R, 8).

#### Responsible

The physicians perceived expectations to assume responsibility for patient safety as part of their professional role, which includes being in charge of medical decision making. However, they provided many examples of various forms of organizational barriers that create difficulties for them to live up to such expectations. They recognized a disparity between behaviours associated with this patient safety responsibility and the conditions required to achieve safe care. This seemed to generate a sense of disappointment among many physicians who felt they cannot contribute sufficiently to patient safety.

The physicians were intent on carrying out their work diligently and efficiently despite the recognized challenges. However, they were aware that their actions or decisions might not always be in accordance with the ideals of safe care delivery, thus jeopardizing patient safety.

Often you have to discharge patients too early - sometimes you have the feeling that if this would have been my relative I wouldn’t have sent the patient home, but somehow there’s no choice. (R, 7).

The responsibility for patient safety was not always proportional to the training they felt was needed to maintain standards for safe care. Work introductions and education in reporting adverse events were inferior or non-existent, according to the physicians. Physicians in education (internship and residency) argued that their responsibility for patient safety was sometimes unrealistically high given the circumstances.

The introductions differ a lot. As a new physician in a department you might have half a day to learn the routines and get your introduction. A nurse has three to four weeks. There are built-in differences in how we work. (C, 12).

One part is the education [concerning reporting system]. Has anyone [physician] even seen the incident reporting system? Do we know how to use it. Do we know why we do it [report incidents]. (R, 8).

Although the physicians felt they were trusted to uphold patient safety standards, they noted that management’s patient safety-related decisions or policies, e.g. statements regarding the importance of patient safety, often did not provide much support in their everyday work with patients, at the front line of health care. Some physicians believed that trust in management might erode if they are assumed to bear responsibility for patient safety without adequate support, something that may have a negative impact on their engagement in patient safety efforts.

Individual managers, the immediate manager - their attitudes in dealing with these things [safety issues] - I think that’s also a key factor. (R, 8).

But, if the top management says that patient safety is important, that patients shouldn’t fall from beds, you have to make analyses, why do you have complications? Then we do it [work with patient safety issues] of course and we want to work with it. But we need an approved assignment for it. (C, 14).

### Theme: The integrated yet independent physician

The integrated yet independent physician theme describes physicians’ values and norms associated with expectations to simultaneously be an autonomous “soloist” and an actively contributing team player who is an integral part of the multi-professional teams in health care.

#### Autonomous

The physicians described expectations of acting independently due to the profession’s special authority over specific areas of expertise and their high degree of status in health care. They felt that they had the discretion to decide about their involvement in management-led initiatives because they considered patient care to be their first and foremost obligation. The physicians valued their freedom to control their work, although they recognized that both positive and negative consequences for patient safety might accrue from their independence.

Many statements by the physicians suggested a somewhat questioning and sceptical attitude to management decisions and policies, including those related to patient safety. They usually wanted rational arguments from the management if they were to commit themselves to something other than patient work. They considered themselves to be best equipped to decide on prioritization of their activities.

There are many different ways of doing things, what’s decided from management is almost never the easiest. In a stressed organization the fastest way is chosen - even if you take a shortcut, simply because it makes daily work function in a better way. (R, 7).

There is sometimes resistance to various procedures and checklists as they are not put in context. The checklist for operations is simple, concise and it is quite easy to understand the benefits of it; must have a clear purpose when you should do things as a routine. (C, 13).

A certain degree of scepticism concerning adverse event reporting was conveyed, because the physicians felt it has an individual focus on errors rather than being used for the purpose of improving the overall system. Reporting was described by some physicians in terms of punishment and blame, and they seemed unconvinced that it was an opportunity for learning to achieve safer care. They wanted to determine which and when adverse events should be reported even if they recognized that it might deviate from existing guidelines or instructions about reporting.

If I report an incident I have to take time from the patients. Long term, it’s obviously better to make the incident reports, because then you can make a change that will benefit all patients. But it’s hard to find the time. (I, 4).

I think there are a lot of incidents that don’t lead to anything ... ridiculous things that might not mean anything for the patients, don’t waste time on those. (C, 14).

Autonomy also expressed itself among the physicians in terms of wanting to be self-sufficient professionals who demonstrate confidence in their work and abilities, which is incompatible with asking for help with specific issues or having too many questions. While they did recognize the importance of training and having support from more experienced physicians, admitting to having insufficient knowledge or skills seemed to contrast with the physicians’ self-image as authoritative, self-reliant professionals who always act with certainty and decisiveness. Some physicians believed that the expectations for independence could yield feelings of ambiguity or vulnerability, which could potentially challenge patient safety.

You’re very dependent on your consultant. If you feel ... I might be worried if I feel it is ... the consultants of course have much better knowledge than me, but sometimes when you feel that they do not take the unit work so seriously […] I become stressed and anxious because I do not feel I want to have it all on me. […] it’s an incredible comfort to know that the consultant knows [the patients], and has maybe also been at the unit during the week. (I, 1).

[I] think for sure that wrong decisions can be made at several times because there’s no feedback and contact with a consultant on call is to be kept to a minimum. (R, 6).

#### A team player

The physicians expressed that they are expected to be involved in team work and be a member of multi-professional teams. They described that when their role in the team was clear and well defined, it could provide them with a comfortable, secure environment in which it was easy to ask questions, including those that could be important for patient safety. However, they also described situations where they did not feel like a full member of the team. This produced feelings of uncertainty or of being ignored, which could have a negative impact on patient safety.

Physicians’ team involvement was compared with that of registered nurses, who usually work in a specific unit and therefore know each other well and have been able to develop efficient collaboration and work routines. In contrast, physicians work in many different units, making it difficult to develop closer working relationships and keep up to date with changes in routines and daily tasks.

[…] registered nurses working at their unit and stationed there, know all the routines. Physicians go all over and are supposed to manage all the units, but may not do so. You don’t know the procedures and everything that goes on there [at the specific unit]. You’re left feeling insecure because you don’t know more than that […]. (R, 5).

Always new staff groups, so the group dynamic… there are always new changes. You think at day number five [in the same unit]: I hardly recognize you, have we said hello before? That makes it a little more insecure. (I, 3)

The physicians valued collaboration with registered nurses and believed this professional group was crucial for patient safety. Many physicians expressed a need to have competent registered nurses in their teams. According to some physicians, a high turnover of registered nurses can jeopardize patient safety and have a negative influence on their own situation.

We have a very good cooperation with registered nurses and great confidence in their judgements. (R, 6).

I often feel proud of the RNs working in my department, it’s a pretty tough unit to work in I think. Meeting other nurses working as a consultant on call in other departments, I think our nurses are more driven and more active. I think they are very capable. (C, 15).

Being a team player and communicating across professional boundaries in health care was recognized as potentially beneficial for patient safety. However, despite the assumption that different professions work synergistically in practice, the physicians noted that professions are not inclusive, with everyone seemingly struggling with their own issues. Still, the physicians argued for increased inter-professional collaboration and communication although they commented that not all physicians might appreciate such a development because it could challenge their sense of authority as it forces them leave their comfort zone.

We haven’t got the same daily work […], causing us to see things from very different perspectives and we don’t communicate these visions; it’s real easy then to blame other groups when you don’t have personal contact. It’s easy to perceive your own reality as heavy and burdensome, while you think of others as being easier […]. Poor understanding between staff groups due to lack of meeting points. (R, 9).

[I] think there’s quite a lot of resistance, especially from physicians, for meetings together [with RNs]; it should be structured from the beginning to prevent the physicians from saying ‘That was bad, we won’t take part again.’ (R, 7).

## Discussion

The aim of this study was to explore physicians’ professional culture in terms of values and norms that may have relevance for patient safety in Sweden. The competent physician theme describes expectations for physicians to be infallible and responsible for patient safety, whereas the integrated yet independent physician theme concerns expectations to be autonomous but also a team player. We found several aspects of the physicians’ culture that could potentially have an impact on patient safety and efforts to achieve safer care.

The physicians expressed values and norms concerning expectations for infallibility of physicians as a professional group. However, they seemed to view this as a burden and described the silence that results from reluctance to discuss any errors they make. All humans make errors, but errors create important learning opportunities for improved patient safety [20]. The change process that an individual who makes an error must deal with to achieve learning has been described [21] as follows: first a confrontation with an experience that did not fit their previous assumptions about self; next resistance (ignoring or reinterpreting the event); followed by validation when the truth is recognized; and finally integration, which allows the previous and new knowledge about self to be synthesized for new learning. This individual learning must be converted into organizational learning for more substantial impact on patient safety in a health care organization. However, research suggests that learning in relation to errors tends to be narrowly focused on the individual most closely involved in the event [22, 23]. Organizational learning requires transparency and open communication, which is difficult to achieve in a culture of silence, in which errors are seen as proof of incompetence [24].

The system perspective has become the “espoused theory” of patient safety today, i.e. most errors are considered to be due to faulty systems, latent process failure and other conditions that lead people to make errors [25]. Still, our findings suggest that many physicians have a strong emphasis on individual responsibility and accountability, which means that the “theory-in-use” among many physicians is more individualistically oriented. Other studies have described a “culture of perfection” among physicians, which means that errors are viewed as personal failures [22, 26]. In their training to be physicians, they are taught to abide by the credo “First, do no harm”, which appears to be a view of themselves that continues from training into practice [23].

The physicians interviewed for our study expressed values and norms that showed that they wanted to be accountable for patient safety. This responsibility is likely associated with physicians’ strong patient focus; their primary loyalties are to their patients [27]. However, the physicians perceived that they did not have adequate management support to take on this role to the extent to which they aspired. Some of the physicians felt deserted at the front line of health care. The importance of knowledge and training for management has been emphasized as means of getting managers in health care to devote more attention to patient safety [28]. Initiatives such as Patient Safety Walkrounds are intended to engage managers in patient safety issues [29, 30].

The values and norms associated with being autonomous could create goal conflicts with regard to efforts for improved patient safety, something that the physicians in our interviews seemed to be fully aware of. They tend to view organizational goals as secondary to their own patient focus and medical decision making. Physicians typically consider their clinical work to be so complex that they need to be able to exercise their professional judgement, which may exclude compliance with management goals or recommendations, including those that concern patient safety [31]. Research has shown that physicians are more sceptical of adverse events reporting than other professional groups in health care [23] and that they have limited knowledge concerning the adverse event reporting process [26]. Physicians can be both gatekeepers and champions with regard to patient safety and other quality improvement efforts [32]. It is important to involve physicians in patient safety efforts at different levels, and to engage them in the development and implementation of guidelines for improved patient safety.

Despite the fact that the culture of medicine traditionally has emphasized autonomy and individualism rather than teamwork [16], the physicians in our study expressed values and norms associated with expectations to be inter-professional team players. However, they said it was difficult to live up to this role, suggesting a conflict between their traditional role as “soloists” who work independently and their role as team members. They recognized that their circumstances are very different from those of the registered nurses, who work more closely together. This allows the registered nurses to develop a close collaborative practice that is impossible to achieve for physicians whose work tasks and duties make it difficult to fully belong to inter-professional teams.

Teamwork and well-functioning communication are commonly described as key factors in achieving improved patient safety, and there is evidence that inter-professional team collaboration facilitates positive results and outcomes in quality improvement efforts [15]. Physicians in our interviews expressed difficulties in achieving a shared view, e.g. with regard to goals in patient care, because of different work situations and limited opportunities for communication between professionals. However, research has also pointed to numerous barriers to engaging physicians in collaborative practice, including conflicts with regard to issues of accountability, with physicians usually viewing themselves as being accountable for patient care, a notion that can be challenged by team members from other professional groups. There can also be problems as a result of a lack of understanding of each other’s roles and the scope of practice of other professions [33]. The importance of inter-professional teamwork is recognized in the education and training of the health care professions in Sweden. It is questionable whether this facilitates the development of shared values and norms across different professional groups, still the net effect still is that physicians and nurses cannot “walk in each other’s shoes” because the cultural differences are too considerable [34].

Knowledge about physicians’ norms and values of relevance for patient safety is potentially important for health care development. The results of this study imply that improved inclusion of physicians in team work may influence patient safety. It also shows that physicians’ reluctance to report incidents and their discretion to decide about their involvement in management-led initiatives can be seen as a reflection of the prevailing professional culture. This knowledge may be used to facilitate a more open dialogue about errors and mistakes, offering a means to influence the patient safety culture and achieve improved patient safety in a longer time perspective.

### Limitations

This qualitative study has some shortcomings that must be considered when interpreting the results. The themes and categories described are not intended as an exhaustive list; other studies may yield different aspects or emphasize other priorities. Instead of statistical generalization, we sought analytical (also referred to as theoretical) generalization, by comparing our findings with other studies concerning physicians’ culture and patient safety. To enhance the transferability of the results, we have provided information on the study participants, interview procedure and analysis procedure as thoroughly as possible [35].

Previous experiences and knowledge within the research team may have influenced the study findings. To improve conformability, the themes found in the analysis were reviewed in relation to the entire data. Furthermore, the research team was multi-disciplinary, consisting of a registered nurse with experience in clinical patient work as well as patient safety research (MD), a physiotherapist who has conducted numerous patient safety studies (SC), an experienced implementation and patient safety researcher (PN) and a physician with a long history as a patient safety researcher and policy maker (HR). The professional culture among physicians is complex and this study has focused only on aspects that have relevance for patient safety.

## Conclusions

This study shows that there are several aspects of physicians’ culture that may have relevance for patient safety. Expectations of being infallible reduce physicians’ willingness to talk about errors they make, generating a “culture of silence” that limits opportunities for learning from errors. The autonomy of the physicians is associated with expectations of acting independently and the physicians use their decisional latitude to determine the extent to which they engage in patient safety-enhancing initiatives and activities. The physicians perceived that organizational barriers make it difficult to live up to expectations to assume responsibility for patient safety. Similarly, expectations of being part of multi-professional teams were deemed difficult to fulfil, potentially inhibiting communication of importance to achieve improved patient safety. The overall findings point to the relevance of viewing physicians’ values, norms and expectations in terms of a distinct subculture in health care. This implies that ambitions to influence the patient safety culture by imposing a single, unitary perspective of the organizational culture are unlikely to succeed. It is important for health care managers, policy makers and researchers to recognize the implications of a multi-faceted perspective on the culture of health care organizations, including the physicians’ professional culture, in the effort to improve patient safety.

## Abbreviations

C: Consultant
I: Intern
R: Resident
